# Survey of Infectious Diseases and Infection Prevention Practitioners on Diversity, Equity, and Inclusion Experiences

**DOI:** 10.1017/ash.2024.206

**Published:** 2024-09-16

**Authors:** Christopher Evans, Erika Kirtz, Rekha Murthy, Cedars-Sinai, Jasmine Marcelin, Zanthia Wiley

**Affiliations:** Tennessee Department of Health; University of Nebraska Medical Center; Emory

## Abstract

**Background:** Incorporating diversity, equity, inclusion, and justice into healthcare ensures equitable opportunity to achieve optimal health. Infectious diseases, antimicrobial stewardship, and infection prevention teams rely on consultative recommendations to improve patient care which may be influenced by implicit and explicit biases of the recipient treatment teams. Little is known about how race, ethnicity, and other characteristics impact stewardship and infection control recommendations. **Methods:** A survey of infectious diseases, antimicrobial stewardship, and infection prevention practitioners was developed through the Society of Healthcare Epidemiology of America (SHEA) Antimicrobial Stewardship Committee. The survey was sent electronically to members of the SHEA Research Network and was promoted to attendees of two sessions at IDWeek 2022 and SHEA Spring 2023. Survey questions included demographics, awareness of (and participation in) unconscious bias and microaggression training at their institutions, antibiotic prescribing bias observations, and perceptions of how race, ethnicity, and other characteristics have influenced participants' antimicrobial stewardship and infection prevention recommendations. Descriptive statistics were performed using SAS V.9.4 . **Results:** Among 175 survey respondents, 75% (n=129) were White, 16% (n=27) were Asian, 4% (n=7) were Black, 85% (n=150) were non-Hispanic, 5% (n=8) were Hispanic, and 3% (n=5) reported ethnicity as multiethnic. 76% of respondents identified as female, and 2% as non-binary or gender-fluid. 29% of respondents had a medical degree, 12% had a nursing degree, 7% had a pharmacy degree, and 52% had a degree listed as other (7% had a PhD, 23% had an MPH/MSPH degree, and 15% had an MS degree). 65% and 49% of respondents had participated in unconscious bias and microaggression training, respectively. 18% (n=22) of White respondents, 43% (n=3) of Black respondents, and 30% (n=8) of Asian respondents reported witnessing antimicrobial prescribing influenced by race, ethnicity, or other characteristics. 17% and 15% of respondents felt that their antimicrobial stewardship and infection prevention recommendations, respectively, had not been accepted due to their race, ethnicity, gender identity, or other personal identifiers. **Conclusion:** This survey showed demographic characteristics of professionals working in infectious diseases and their perceptions of how certain aspects of their identity have influenced their recommendations. Differences between racial groups were observed in how frequently respondents witnessed inequities in antimicrobial prescribing, and many respondents felt their recommendations had not been accepted due to their identity. A limitation of this analysis is that few Black individuals completed the survey, which makes comparisons by race difficult; however, the respondents were consistent with SHEA membership demographics.

